# Rehabilitation, Using Guided Cerebral Plasticity, of a Brachial Plexus Injury Treated with Intercostal and Phrenic Nerve Transfers

**DOI:** 10.3389/fneur.2017.00072

**Published:** 2017-03-03

**Authors:** Lars B. Dahlin, Gert Andersson, Clas Backman, Hampus Svensson, Anders Björkman

**Affiliations:** ^1^Department of Hand Surgery, Skåne University Hospital, Malmö, Sweden; ^2^Department of Translational Medicine – Hand Surgery, Lund University, Malmö, Sweden; ^3^Department of Clinical Neurophysiology, Skåne University Hospital, Malmö, Sweden; ^4^Department of Clinical Sciences in Lund – Neurophysiology, Lund University, Lund, Sweden; ^5^Department of Hand Surgery, University Hospital of Northern Sweden, Umeå University, Umeå, Sweden; ^6^Department of Surgical and Perioperative Sciences, Section for Hand and Plastic Surgery, Umeå University, Umeå, Sweden

**Keywords:** brachial plexus injury, nerve transfer, intercostal nerve, phrenic nerve, electromyography, cerebral plasticity, guided plasticity, rehabilitation

## Abstract

Recovery after surgical reconstruction of a brachial plexus injury using nerve grafting and nerve transfer procedures is a function of peripheral nerve regeneration and cerebral reorganization. A 15-year-old boy, with traumatic avulsion of nerve roots C5–C7 and a non-rupture of C8–T1, was operated 3 weeks after the injury with nerve transfers: (a) terminal part of the accessory nerve to the suprascapular nerve, (b) the second and third intercostal nerves to the axillary nerve, and (c) the fourth to sixth intercostal nerves to the musculocutaneous nerve. A second operation—free contralateral gracilis muscle transfer directly innervated by the phrenic nerve—was done after 2 years due to insufficient recovery of the biceps muscle function. One year later, electromyography showed activation of the biceps muscle essentially with coughing through the intercostal nerves, and of the transferred gracilis muscle by deep breathing through the phrenic nerve. Voluntary flexion of the elbow elicited clear activity in the biceps/gracilis muscles with decreasing activity in intercostal muscles distal to the transferred intercostal nerves (i.e., corresponding to eighth intercostal), indicating cerebral plasticity, where neural control of elbow flexion is gradually separated from control of breathing. To restore voluntary elbow function after nerve transfers, the rehabilitation of patients operated with intercostal nerve transfers should concentrate on transferring coughing function, while patients with phrenic nerve transfers should focus on transferring deep breathing function.

## Introduction and Background

A brachial plexus injury (BPI) is a devastating injury, which can result in severe and permanent neurologic impairment and disability of the upper extremity. Recovery after surgical reconstruction using nerve grafting and nerve transfer procedures for BPI is a function of peripheral nerve regeneration and adaptations within the central nervous system, i.e., plasticity. The plastic capacity of the brain opens possibilities, where plasticity can be guided to substitute or improve functions that have been damaged or lost, i.e., guided plasticity (1). Nerve, muscle, or tendon transfers are procedures that require a plasticity to function, and this is important to take into consideration during rehabilitation after such a procedure (2–4).

Intercostal nerves are commonly used to reinnervate muscles after a BPI with avulsion of spinal nerve roots (5–7); where two intercostal nerves should be enough for reinnervation of a muscle (8). Intercostal nerves can be harvested without any residual problems, e.g., pulmonary dysfunction (9). In contrast, the phrenic nerve, innervating the diaphragm, should only be used as a second alternative as a nerve transfer for muscle reinnervation because of potential pulmonary dysfunction (9–11). An important question is how patients with these two nerve transfers should be rehabilitated to transfer control of the original nerve function to a new function, for example, elbow flexion. Here, we describe a patient with a BPI, who was initially treated with a transfer of intercostal nerves to the musculocutaneous nerve to acquire elbow flexion, but due to insufficient regain of active elbow flexion a second procedure, i.e., a free gracilis muscle transfer reinnervated by a phrenic nerve transfer, was performed.

Our aim is to visualize, through electromyography (EMG), the different activation patterns of the biceps and gracilis muscles by coughing and deep breathing, respectively; observations that are relevant for how patients with such nerve transfers can be rehabilitated after surgery.

## Case Report

A 15-year-old boy sustained a severe BPI with a complete loss of motor and sensory function corresponding to the brachial plexus in the dominant, right, arm after a motorcycle accident. There was no blunt trauma to the patient’s chest according to the history of the patient, and the results from the trauma-computer tomography (CT) just after the accident did not indicate any injury to the chest wall or to the lungs. The patient also had a Horner syndrome. The extent of the BPI was confirmed after a week by magnetic resonance imaging (MRI) and CT-myelography as well as at surgical exploration 3 weeks post-injury; showing avulsion of nerve roots C5–C7 and injuries to the spinal roots C8–T1 (i.e., intraoperatively not ruptured, but in continuity). No further action was done concerning the two lower roots. At the surgical reconstruction, 3 weeks after the injury multiple nerve transfers, focusing on restoring shoulder and elbow function, were done: (a) the terminal part of the accessory nerve was transferred to the suprascapular nerve, (b) the second and third intercostal nerves transferred (the upper intercostal *via* two radial nerve grafts) to the axillary nerve, and (c) the fourth to sixth intercostal nerves were transferred (the two lower *via* three radial nerve grafts) to the musculocutaneous nerve. Postoperatively, he trained under the supervision of a physiotherapist with experience in rehabilitation of patients with BPI.

Twenty-one months after surgery, he had voluntary activation in the infra- and supraspinatus muscles [Medical Research Council (MRC) grade 3], but insufficient function in the biceps muscle (i.e., M1–2). Therefore, the contralateral gracilis muscle was transferred, as a free muscle graft and attached to the coracoid process and to the distal parts of the biceps tendon 25 months after the first procedure. The gracilis muscle was directly reinnervated, through end-to-end repair to the distal part of the specific nerve branch originally innervating the transferred gracilis muscle, using the phrenic nerve that was harvested *via* an open thoracotomy. The patient had postoperative complications with pneumonia on the left side (X-ray examination; spiral-CT also to exclude pulmonary emboli) and a delayed wound healing in the upper part of the incision on the right upper arm, which was treated with antibiotics, revisions with split skin graft, and vacuum-assisted closure therapy, where after these conditions were completely healed. Ultrasound investigations with color-laser-Doppler at 6 and 8 weeks showed a viable gracilis muscle with detectable blood vessels. Postoperative training was initiated 6 weeks after the operation.

**Figure 1:**
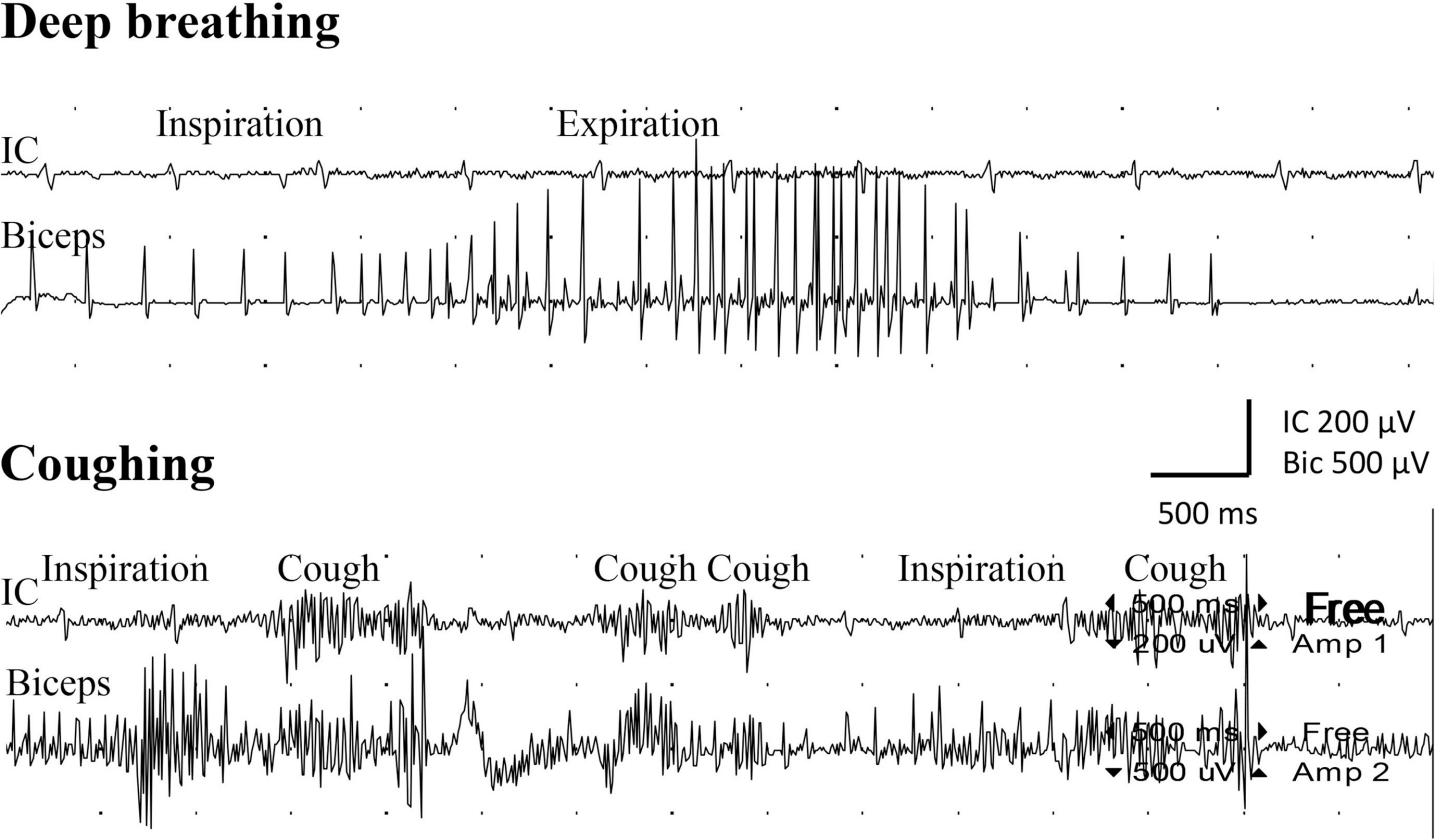
**Electromyogram recorded from the intercostal muscles (IC), distal to level of the transferred intercostal nerves (i.e., around eighth costal level; surface electrodes), as well as from the biceps muscle (biceps; needle electrode) after the second surgery**. Recordings were done during deep breathing (upper panel) and during coughing (lower panel).

**Figure 2:**
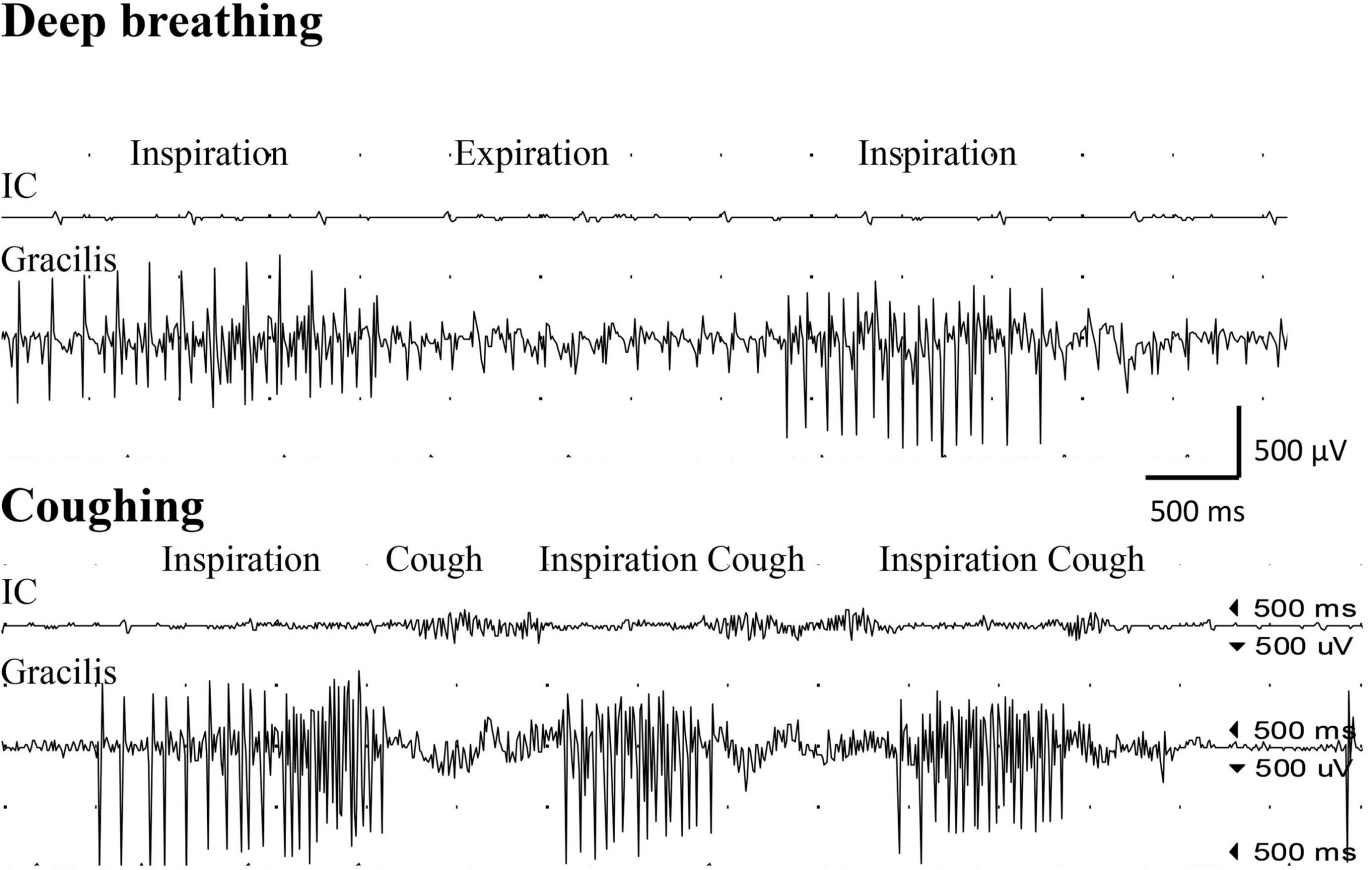
**Electromyogram recorded from the intercostal muscles (IC), distal to the transferred intercostal nerves (i.e., around eighth costal level; surface electrodes), as well as from the gracilis muscle (gracilis; needle electrode) after the second surgery, where the gracilis muscle was transferred as a free muscle graft and reinnervated by the phrenic nerve**. Recordings were done during deep breathing (upper panel) and during coughing (lower panel).

**Figure 3:**
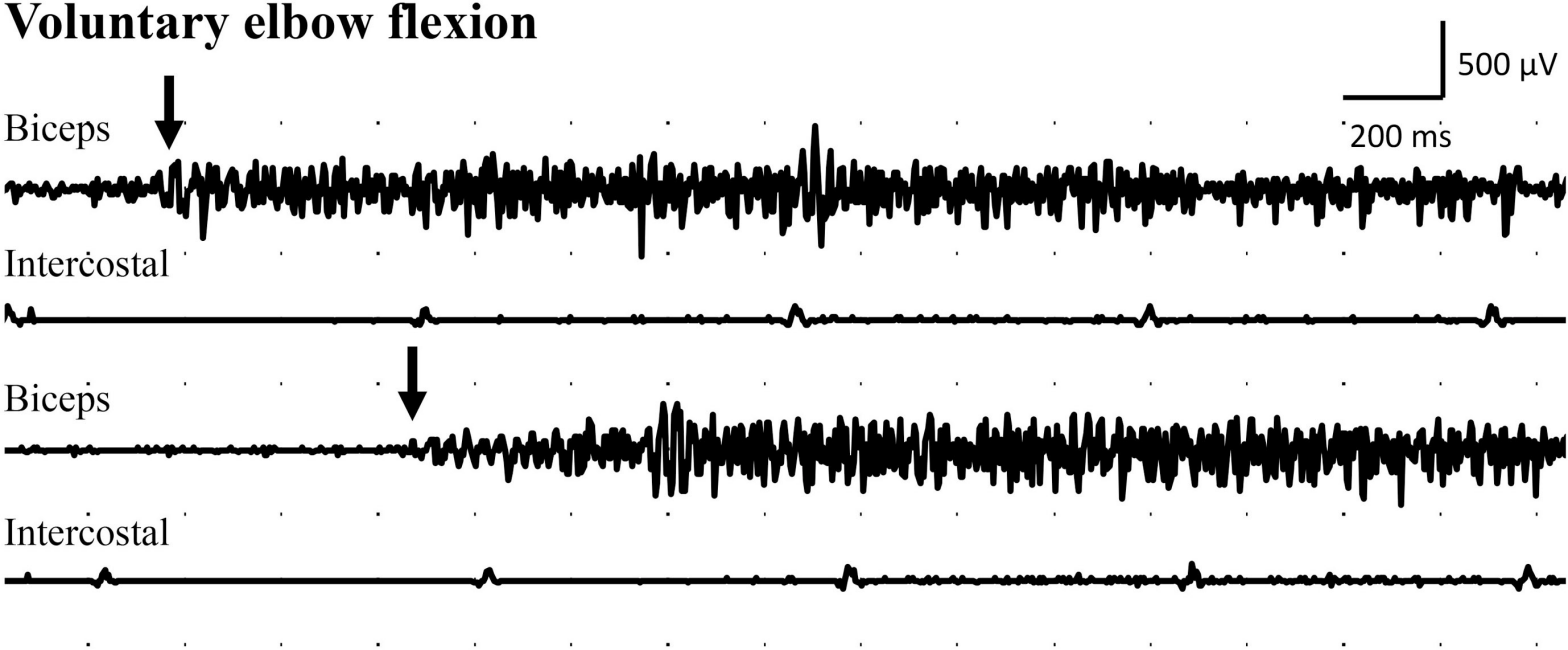
**Electromyogram recorded from the intercostal muscles (intercostal), distal to the transferred intercostal nerves (i.e., around eighth costal level; surface electrodes), as well as from the biceps muscle (biceps; needle electrode) after the second surgery**. Recordings were done during voluntary elbow flexion (arrow indicates start of the voluntary flexion).

Examination with EMG after clinical signs of reinnervation, i.e., 1 year after surgery, showed extensive denervation activity in the biceps muscle with few neurogenic altered activated motor units in the muscle through activation of the intercostal nerves (i.e., essentially with coughing but also with deep expiration, Figure 1), although still suboptimal strength (M1–2). The transferred gracilis muscle, innervated by the phrenic nerve, showed no denervation activity, but was rhythmically activated (a large number of motor units) during the inspiration phase of deep breathing (not with coughing; Figure 2), and by voluntary elbow flexion (weak M3). Voluntary flexion of the elbow (weak M3; up to 30 degrees of flexion) elicited clear activity in the biceps/gracilis muscles, with minor activity in intercostal muscles (shown for biceps in Figure 3). The intercostal muscles showed activation during coughing, while the modulation during normal breathing was hardly detectable with surface electrodes. At the final follow-up, there was no muscular activity at all, observed or palpated, in the brachioradialis muscle (M0), i.e., the elbow function was entirely caused by the biceps and gracilis muscles. He had no supination induced by activity in the supinator muscle, but supination (M3) was observed to be performed by the biceps/gracilis muscles as one would expect due to their insertion at the tuberosity of the proximal part of the radius. No function was observed in the pronator muscle to compensate for the supination. Clinical examination also revealed some finger (i.e., flexor digitorum superficialis/profundus muscles) and thumb (i.e., flexor pollicis longus muscle) activity [i.e., M3–4(−)], but without any real function in activity of daily living. No further surgery was performed. Rehabilitation was terminated in agreement with the patient 2.5 years after the latest surgical procedure, although he still did not show optimal elbow function.

## Discussion

In the present case, the initial procedures, after careful preoperative evaluation of the injury with MRI and CT-myelography (12), included different nerve transfers to improve/restore shoulder and elbow function (13). The preoperative investigations, as well as intraoperative findings, indicated continuity of the C8 and Th1 and lower trunk, but without clinical function, and no further action was taken concerning this part of the brachial plexus, including attention to elbow extension. Some finger and thumb function, although not functional in daily activity, had returned at the final follow-up, indicating that C8 and Th1 roots were indeed not avulsed at injury. We cannot explain the presence of the Horner syndrome in spite of the lack of avulsed C8 and Th1.

Stabilization of the shoulder was achieved with the first two mentioned transfers at the initial procedure, while the conventional transfer of three intercostal nerves to the biceps brachii muscle was insufficient to restore elbow function (M1–2) in the present case (8). In the second procedure, it was necessary to do a free gracilis muscle transfer with the intention to improve elbow flexion (14–16). Because the intercostal nerves had been used in the first surgical procedure, we chose to reinnervate the transferred gracilis muscle through the phrenic nerve (17, 18), which can be done without affecting pulmonary function although intercostal nerves previously have been used (19). The phrenic nerve was harvested and transected close to the diaphragm through an open thoracotomy. The possibility of a contralateral C7 transfer was discussed (20), but declined for several reasons, including the patient’s own opinion. The phrenic nerve was sutured directly end-to-end to the distal nerve supplying the gracilis muscle with the intention to have a short distance for the regenerating nerve and thus a more rapid reinnervation of the muscle (9). In this way, it was also possible to avoid nerve grafts, although considered to be not necessary in phrenic nerve transfer to musculocutaneous nerve (21). The intrathoracic part of the phrenic nerve is rather thin and by relocating the phrenic nerve extensively, as was done in the present case, there may be a risk that the intraneural blood supply can be jeopardized. However, it is known that nerve grafts, and also a thin phrenic nerve, can survive through diffusion before revascularization. This was obviously the case here since the results from the EMG showed that the axons survived and had grown into the gracilis muscle with functional reinnervation. The reinnervation of the present transferred gracilis muscle, noted clinically and by EMG, is in agreement with an earlier report examining gracilis muscle transfers in a large population, where those patients were reconstructed with a similar surgical delay as well as had a similar postoperative follow-up and time for reinnervation (22). Interestingly, around 35% of their patients had a similar recovery (i.e., M3), based on MRC scale, as the present patient, while only 26% had M4 (22). In the present case, the intercostal nerves and the phrenic nerve were used to restore elbow function at two different time points with the purpose to reinnervate the elbow flexors through the musculocutaneous nerve and through the branch innervating the transferred gracilis muscle; thus, a synergistic function could be achieved, which is an advantage in view of the acting cerebral plasticity. Previous data indicate that the two nerves should not be used simultaneously to restore elbow extension and flexion, respectively; thus, with an antagonistically intended function (19).

The clinical outcome following complex brachial plexus reconstructions, like the present one, is not only dependent on regeneration of nerve fibers into the target muscle, but also highly dependent on adaptations within the central nervous system, i.e., plasticity. This was observed in the present case since the patient also could independently, without coughing or deep breathing (i.e., “respiratory standstill”), activate both the biceps and the gracilis muscles by voluntary elbow flexion although it was not functional [i.e., only weak M3 (3)]. Thus, an initial coughing and deep breathing function of the intercostal and phrenic nerves, respectively, had been transferred to voluntary elbow flexion through cerebral plasticity.

A nerve transfer will always induce a cerebral reorganization as well as alteration of respiratory spinal descending inputs to thoracic motoneurons (23). We did not investigate the influence of trunk flexion in the rehabilitation process. Trunk flexion can activate the biceps muscle through the intercostal nerves as presented by Chalidapong et al. in a larger study (24). Higher amplitude was measured in the reinnervated elbow flexor muscles at EMG in that study, but only 6/32 patients preferred trunk flexion to activate flexor muscles of the elbow; most of the patients preferred other techniques alone or in combination to flex the elbow (24). It is imperative that the team treating patients with BPI reconstructed with muscle and/or nerve transfers has a good knowledge in neuroscience in order to understand the cerebral changes caused by the injury and by the surgical reconstruction. With this knowledge at hand, it is possible to individually tailor the rehabilitation and physical therapy protocols using guided plasticity in order to achieve the best possible clinical outcome (1).

### Limitations of the Study

During normal breathing, expiration is a passive process. Thus, one would not expect any activation during expiration in a muscle where a nerve is transferred. However, in deep breathing, the expiratory phase can be an active process with the purpose to speed up such a phase. Hence, some EMG activation can be expected during deep breathing, which was observed in the biceps muscle as indicated in Figure 1. In the present case, we observed little activity during normal and deep breathing, with EMG, from the intercostal muscles, when one would expect that the quiet and passive expiration would not result in any activity at all. We have no explanation for this observation, but during coughing, bursts of EMG activity were recorded from the intercostal muscles as expected. The intercostal muscles showed activation during coughing, while the modulation during normal breathing was hardly detectable with surface electrodes.

## Concluding Remarks

The present data indicate that intercostal nerves, which was used in the first surgical procedure since there were no concomitant chest trauma (25), support important function as coughing, while the phrenic nerve is crucial for deep breathing. We suggest that the training of patients, in whom transfer of intercostal nerves has been performed, should concentrate to transfer coughing function to the biceps brachii muscle, which is in contrast to a previous study, suggesting trunk flexion during training of the new function in order to facilitate elbow flexion (24). In contrast, patients with a phrenic nerve transfer, which can be performed through harvesting the phrenic nerve *via* a thoracotomy for direct end-to-end attachment to the distal nerve end of the target muscle, should focus on transferring deep breathing function to the reinnervated muscle.

## Ethics Statement

The patient was treated with conventional clinical procedures in the health care sector not requiring any ethical approval. We have informed consent from the patient to publish the history and treatment, which is stated in the patient journal folder.

## Author Contributions

LD: overall responsible for diagnosis and clinical treatment of the patient; concept of message, draft, and revision and finalization of the manuscript; GA: responsible for neurophysiological examination and draft of the manuscript; CB: responsible for treatment of the patient and draft of the manuscript; HS: draft of the manuscript; AB: draft and finalization of the manuscript.

## Conflict of Interest Statement

The authors declare that the research was conducted in the absence of any commercial or financial relationships that could be construed as a potential conflict of interest.
